# Efficient and Air-Stable Planar Perovskite Solar Cells Formed on Graphene-Oxide-Modified PEDOT:PSS Hole Transport Layer

**DOI:** 10.1007/s40820-017-0140-x

**Published:** 2017-03-17

**Authors:** Hui Luo, Xuanhuai Lin, Xian Hou, Likun Pan, Sumei Huang, Xiaohong Chen

**Affiliations:** Engineering Research Center for Nanophotonics and Advanced Instrument, Ministry of Education, and School of Physics and Materials Science, East China Normal University, No. 3663 North Zhongshan Rd., Shanghai, 200062 People’s Republic of China

**Keywords:** Perovskite solar cells, Moisture resistance, Wettability, Stability, Graphene oxide, PEDOT:PSS

## Abstract

As a hole transport layer, PEDOT:PSS usually limits the stability and efficiency of perovskite solar cells (PSCs) due to its hygroscopic nature and inability to block electrons. Here, a graphene-oxide (GO)-modified PEDOT:PSS hole transport layer was fabricated by spin-coating a GO solution onto the PEDOT:PSS surface. PSCs fabricated on a GO-modified PEDOT:PSS layer exhibited a power conversion efficiency (PCE) of 15.34%, which is higher than 11.90% of PSCs with the PEDOT:PSS layer. Furthermore, the stability of the PSCs was significantly improved, with the PCE remaining at 83.5% of the initial PCE values after aging for 39 days in air. The hygroscopic PSS material at the PEDOT:PSS surface was partly removed during spin-coating with the GO solution, which improves the moisture resistance and decreases the contact barrier between the hole transport layer and perovskite layer. The scattered distribution of the GO at the PEDOT:PSS surface exhibits superior wettability, which helps to form a high-quality perovskite layer with better crystallinity and fewer pin holes. Furthermore, the hole extraction selectivity of the GO further inhibits the carrier recombination at the interface between the perovskite and PEDOT:PSS layers. Therefore, the cooperative interactions of these factors greatly improve the light absorption of the perovskite layer, the carrier transport and collection abilities of the PSCs, and especially the stability of the cells.

**Electronic supplementary material:**

The online version of this article (doi:10.1007/s40820-017-0140-x) contains supplementary material, which is available to authorized users.

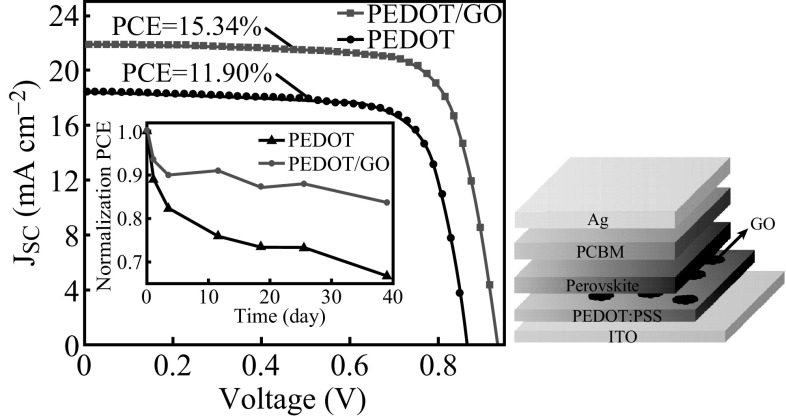

## Highlights

- PSCs (perovskite solar cells) with GO (graphene-oxide)-modified PEDOT:PSS (poly(3,4-ethylenedioxythiophene)-polystyrenesulfonate) exhibit a higher efficiency and better stability.
- GO-modified PEDOT:PSS surfaces exhibit superior wettability relative to PEDOT:PSS.
- Hole extraction selectivity of GO inhibits carrier recombination of PSCs.

## Introduction

Perovskite solar cells (PSCs) have attracted considerable attention because of their low-cost and high-efficiency_ENREF_1. The power conversion efficiency (PCE) of mesoporous PSCs has already reached more than 22.1% [1]. Although a low-temperature processing method has been reported for mesoscopic PSCs using nanoparticles [2], high-temperature (500 °C) processing is still required to produce the most efficient mesoscopic PSCs [3, 4]. Relative to mesoporous PSCs, planar PSCs are expected to consume less energy and save cost, and promise to be a more feasible means of fabricating cells on a flexible plastic substrate, while showing capability of being integrated into tandem solar cells [5–7].

Currently emergent inverted (p-i-n) planar PSCs yield a 20.3% PCE [8], which is almost comparable to that of mesoporous PSCs. Inverted planar PSCs, which typically use a MAPbI_3_-PCBM ([6]-phenyl-C 61-butyric acid methyl ester) bilayer junction, offer some advantages in terms of their simple fabrication and lower hysteresis [9, 10]. Poly(3,4-ethylenedioxythiophene)-polystyrenesulfonate (PEDOT:PSS) which offers a high level of conductivity has been widely applied as a hole-selective material in both polymer solar cells [11–13] and inverted PSCs [5, 14–16]. However, the use of PEDOT:PSS would greatly limit the lifetime and performance of cells due to its hygroscopic nature and inability to block electrons [12]. Therefore, p-type inorganic materials, such as CuSCN [17], NiO [18], and CuI [19], have been proposed as promising alternative to PEDOT:PSS to enhance the stability of PSCs. However, the low conductivity of inorganic materials limits the performance of PSCs. Recently, heavily p-doped LiNiMgO was developed as a hole transport layer (HTL) by Chen et al. [20], allowing PSCs to attain a PCE of 16.2% with an area of 1 cm^2^. However, high-temperature processes are usually used to improve the conductivity of the inorganic hole transport layer, including the LiNiMgO layer. To develop a better hole transport layer with a low-temperature solution process, some hole inorganic material and PEDOT:PSS composites have also been developed to overcome these shortcomings. GeO_2_-doped PEDOT:PSS [21], NiOx-PEDOT:PSS [22], and MoOx-PEDOT:PSS [23] have been demonstrated to be efficient hole-transporting materials for planar PSCs with efficiencies exceeding 15% due to the better coverage of the perovskite films and the hole transport ability.

Graphene oxide (GO) is a graphene sheet functionalized with oxygen-containing functional groups, in the form of epoxy and hydroxyl groups on the basal plane and various other groups, such as carboxyl, carbonyl, phenol, and quinone, typically at the sheet edges [24, 25]. GO and reduced graphene oxide (RGO) have been used as the HTL in PSCs [26] and polymer solar cells [27]. The use of oxygen-containing functional groups of GO as an amphiphilic functional layer improves the wettability of the perovskite precursor solution, which improves the surface coverage and grain uniformity of the perovskite film. However, the poor conductivity of GO due to its high oxygen content makes the performance of cells highly sensitive to the thickness of the GO film [28]. The GO and PEDOT:PSS composite compensates for the drawbacks of single GO and conventional PEDOT:PSS in that it exhibits a higher PCE of 9.7% and a better stability in air compared to that of PSCs with a PEDOT:PSS layer [29]. Li et al. [30] reported on the use of GO as interface modifier, inserted into the interface between the perovskite and a spiro-MeOTAD hole transport layer, thus improving the contact between the perovskite and spiro-MeOTAD layer, by passivating the surface defects in the perovskite film and preventing charge recombination.

In this work, a GO solution was spin-coated onto the PEDOT:PSS surface to form a GO-modified PEDOT:PSS layer. The performance of PSCs based on both unmodified and GO-modified PEDOT:PSS was investigated. It was found that PSCs modified with GO exhibited a higher PCE and better stability in air. The enhanced performance of the GO-modified PSCs is attributed to the superior wettability of the GO-modified PEDOT:PSS surface, retarding carrier recombination, and partial removal of the PSS material at the PEDOT:PSS surface.

## Experimental Section

GO powder was first dispersed in ethanol using a bath sonicator, at a concentration of 1 mg mL^−1^, and then diluted to 30 mg L^−1^. The GO was synthesized from graphite powder using a modified Hummer method [31]. A perovskite precursor solution was prepared by dissolving 1-M methyl ammonium iodide (MAI, 1-Material Inc.) and 1-M lead (II) iodide (PbI_2_, 99.9%, Aladdin Reagents) in a mixture of dimethyl sulphoxide (DMSO, AR 99% GC, Aladdin Reagents) and γ-butyrolactone (GBL, AR 99% GC, Aladdin Reagents) (7:3 v/v). Cleaned ITO-coated glass with a sheet resistance of 10 Ω/□ was treated with ultraviolet ozone for 20 min. PEDOT:PSS solutions (CLEVIOS PVP Al 4083, Heraeus) were spin-coated at 4000 rpm for 1 min to form a 20-nm film, followed by annealing at 140 °C for 20 min. Then, GO was spin-coated onto the surface of the PEDOT:PSS layer at 4000 rpm for 1 min in the atmosphere. Next, the samples were transferred into an argon-filled glove box and retained for 1 h before further spin-coating of the perovskite layer. The CH_3_NH_3_PbI_3_ film was spin-coated onto PEDOT:PSS or GO-modified PEDOT:PSS layers by a consecutive two-step spin-coating process at 2000 and 4000 rpm for 20 and 60 s, respectively. During the second spin-coating step, toluene drop-casting was employed to replace DMSO solvent. The samples were dried at 100 °C for 10 min. After being cooled, a 70-nm PCBM layer was formed on the CH_3_NH_3_PbI_3_ film by spin-coating a solution of PCBM in chlorobenzene (10 mg mL^−1^) at 1200 rpm for 60 s. Finally, a 100-nm Ag electrode was thermally evaporated onto the PCBM layer at a base pressure of 5 × 10^−4^ Pa. The process for producing PSCs with a GO-modified PEDOT:PSS layer is shown in Fig. S1.

The surface morphologies of the samples were investigated by field-emission scanning electron microscopy (SEM, Hitachi S-4800) and atomic force microscopy (AFM, JPK Instruments, Germany). The contact angles were measured with an optical contact angle meter (JC2000D3) at room temperature. X-ray diffraction (XRD) patterns of the perovskite films were measured with a PW3040/60 instrument (Holland Panalytical PRO) using a Cu *K*α radiation source (30 kV, 25 mA). X-ray photoelectron spectroscopy (XPS, ESCALAB 250XI, Thermo Scientific) was performed using Al-*K*α monochromatic radiation. The photoluminescence (PL) spectra were measured using a fluorescence spectrophotometer (FluoroMax-4, HORIBA Jobin–Yvon). The transmission and absorption spectra of the samples were recorded using a UV–Vis spectrophotometer (U-3900, Hitachi). The current density–voltage (*J*–*V*) characteristics were measured using a Keithley model 2440 source meter and a Newport solar simulator system with AM1.5G and 100-mW cm^−2^ illumination. The incident photon to current conversion efficiency (IPCE) over a wavelength range of 300–800 nm was measured using an optical power meter (2936-R, Newport). EIS measurements were obtained in the dark using a PGSTAT 302-N electrochemical workstation (Autolab). Ultraviolet photoelectron spectroscopy (UPS) spectra were measured with a monochromatic He II light source (40.8 eV) and an R4000 analyzer (VG Scienta).

## Results and Discussion

**Fig. 1:**
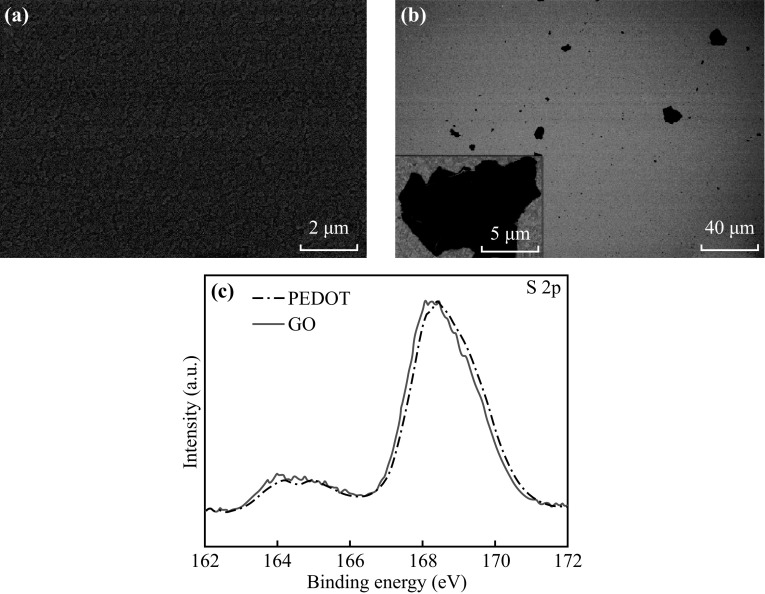
SEM images of PEDOT:PSS modified without **a** and with **b** GO pieces. The *inset* is the high magnification of GO on ITO surface. **c** Normalized XPS spectra S 2p of PEDOT:PSS surface with and without GO modification

Figure 1a, b shows SEM images of the unmodified and GO-modified PEDOT:PSS. The observed GO pieces are scattered over the surface of the PEDOT:PSS layer, indicating that the GO does not form a continuous film. The sizes of the GO pieces range from several hundreds of nanometers to tens of micrometers, as can be seen in the SEM images. The root mean square (rms) of the GO-modified PEDOT:PSS is 2.92 nm, which is slightly rougher than the rms (1.99 nm) of the PEDOT:PSS surface, as shown in Fig. S2. The GO pieces have only a few layers with a typical thickness of about 1.25 nm, as determined from the AFM image, while the relative content of the carbon bound to oxygen (C–O) was found to be 43.8% by XPS analysis according to a previous report [32]. The PSS material of the PEDOT:PSS polymer exhibits hygroscopic properties [33], as borne out by the chemical structure of the PEDOT:PSS, shown in Fig. S3d. PEDOT is insoluble in most solvents, but can be dispersed in water by using PSS as a counterion. The PEDOT phase is surrounded by a thin PSS-rich surface layer, which leads to it having a low conductivity [13, 34]. The ethanol in the GO solution can partly remove the PSS of the PEDOT:PSS surface while spin-coating the GO onto the surface of the PEDOT:PSS layer, which is expected to improve the conductivity of the PEDOT:PSS surface. The *I*–*V* curves of the PEDOT:PSS, PEDOT:PSS treated with ethanol, and PEDOT:PSS/GO films, are shown in Fig. S4. The resistivities derived from the *I*–*V* curves of PEDOT:PSS treated with ethanol and modified with GO are 8.13 and 6.61 Ω m, respectively, which is less than the 12.3 Ω m of the PEDOT:PSS film. The enhanced conductivity of the GO-modified PEDOT:PSS would help to reduce the serial resistance of the cells. The contact angles of the perovskite precursor solution on the PEDOT:PSS layers, modified under different conditions, are shown in Fig. S3a–c. Using a perovskite precursor solution including PbI_2_, MAI, DMSO, and GBL to measure the contact angles is helpful for directly evaluating the wettability of films formed using perovskite. The contact angles of the PEDOT:PSS surfaces with and without ethanol treatment are 14.0° and 21.6°, respectively, indicating that the partial removal of the PSS material by the ethanol treatment adversely affects the wettability of the perovskite precursor. The normalized corresponding high-resolution XPS spectra S 2p of the unmodified and GO-modified PEDOT:PSS surfaces are shown in Fig. 1c. The S 2p peak at a binding energy of 168.4 eV corresponds to the sulfur signal for PSS, while the peak at 164.1 eV corresponds to the sulfur signal of PEDOT. The peaks at 164.1 eV shift slightly toward a lower energy level and slightly increase after the GO solution treatment, indicating that the PSS material of the GO-modified PEDOT:PSS surface was partially removed during the spin-coating of the GO solution [13, 35]. However, despite the partial removal of the PSS material, the contact angle of the GO-modified PEDOT:PSS is only 2.5°, suggesting that the scattered GO pieces exhibit superior wettability with the perovskite precursor solution, which can reduce the nucleation barrier and help form a high-quality perovskite layer [2].

**Fig. 2:**
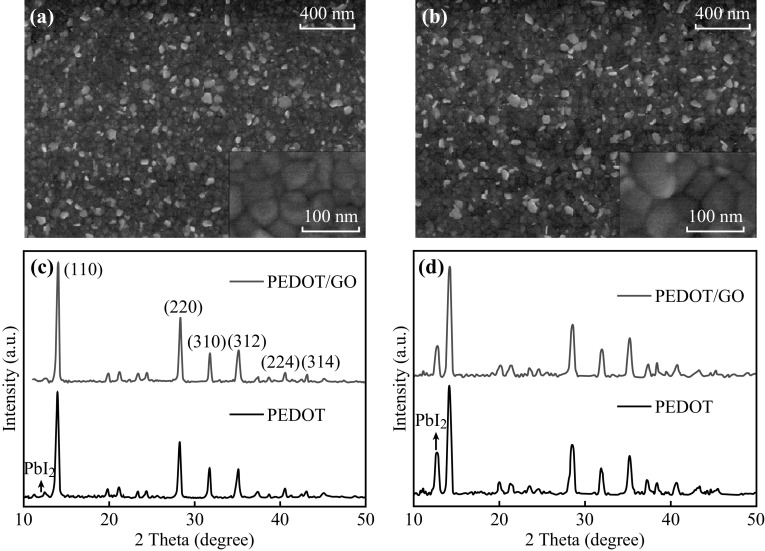
SEM images of perovskite growth on **a** PEDOT:PSS and **b** GO-modified PEDOT:PSS layer. XRD patterns of perovskite film growth on PEDOT:PSS and GO-modified PEDOT:PSS without **c** and with **d** moisture invasion

SEM images of the perovskite films formed on PEDOT:PSS, both with and without the GO particles, are shown in Fig. 2a, b. The surface morphologies of the unmodified and GO-modified perovskite films cannot be clearly distinguished. The perovskite grains with the GO modification seem slightly larger and more uniform in the high-resolution SEM images. Figure 2c shows the XRD patterns of the perovskite films. The strong peaks at 14.02°, 28.41°, and 31.85° correspond to the (110), (220), and (310) planes of the perovskite films. A stronger peak assigned to the (110) plane of the perovskite indicates that the perovskite film with the GO modification has a better morphology and crystallinity, and larger grains, which help to improve the coverage of the perovskite layer with free pin holes [23]. The clear PbI_2_ peak for the unmodified perovskite films can possibly be attributed to the incomplete reaction between the PbI_2_ and CH_3_NH_3_I. The superior wettability of the GO-modified PEDOT:PSS can reduce the nucleation barrier and improve the nucleation density, which may improve the coverage of the perovskite film and the grain uniformity [2]. However, the relatively inferior wettability of the PEDOT:PSS is possibly the reason for the lower-density nucleation sites, which causes the distribution of the perovskite grains to be more scattered such that some large PbI_2_ grains cannot completely react with the CH_3_NH_3_I. It has been widely reported that, in the two-step perovskite layer, the large PbI_2_ grains cannot completely react with the CH_3_NH_3_I [2, 36]. For the mixture of PbI_2_ and CH_3_NH_3_I, the dynamic process of PbI_2_ nucleation and reaction with CH_3_NH_3_I cannot be easily observed or controlled [37]. Given the different intensities of the PbI_2_ peaks, we can conjecture that the process of nucleation and crystallization of the perovskite on GO-modified PEDOT:PSS differs slightly from that on a PEDOT:PSS layer. The disappearance of the PbI_2_ peak for the perovskite layer indicates that the GO-modified PEDOT:PSS surface aids with the formation of more uniform perovskite grains [2]. However, the appearance of a PbI_2_ peak for the perovskite as a result of moisture cannot be excluded because the samples were always exposed to the atmosphere. The PbI_2_ peaks of the perovskite film formed both on GO-modified and on unmodified PEDOT:PSS were significantly enhanced after the samples were exposed to saturated water vapor pressure at room temperature for 1.5 h, as shown in Fig. 2d. However, the intensity ratios between (110) perovskite and PbI_2_ peaks for both the GO-modified and unmodified PEDOT:PSS were 3.88 and 2.33, indicating that the GO-modified perovskite film is more stable, despite the presence of moisture. The more rapid transformation of the perovskite film formed on a pure PEDOT:PSS surface, from dark brown to yellow or pale yellow, can be observed, as shown in Fig. S5, further indicating that the unmodified perovskite film easily decomposes into CH_3_NH_3_I and PbI_2_ [14]. The enhanced stability of perovskite film can be attributed to the better crystallinity and partial removal of the PSS material, which improves the moisture resistance of the perovskite film [14].

**Fig. 3:**
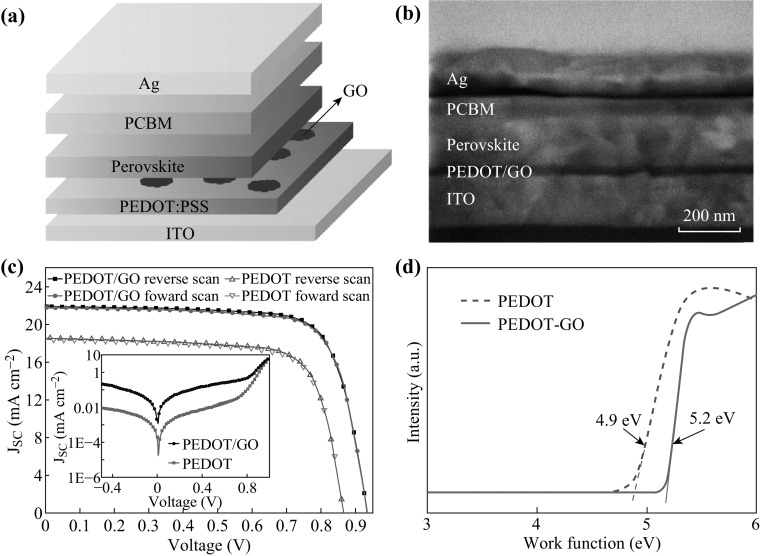
**a** Schematic of cell structure. **b** Cross-sectional SEM image of PSCs with structure of ITO/PEDOT:PSS/GO/CH_3_NH_3_PbI_3_/PCBM/Ag. **c** *J*–*V* curves of PSCs with and without GO-modified PEDOT:PSS. The inset is dark *J*–*V* curves of PSCs. **d** UPS spectra of the PEDOT:PSS surface with and without GO modification

**Fig. 4:**
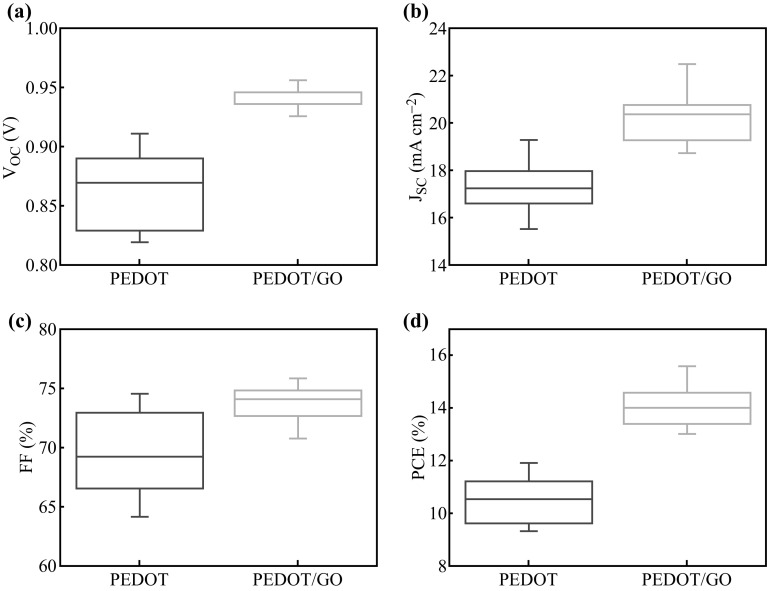
Statistical parameters of PSCs with PEDOT:PSS and GO-modified PEDOT:PSS. Approximately 30 devices of each type were statistically analyzed

To first evaluate the effect of the pure GO film and PEDOT:PSS treated with ethanol on the performance of PSCs, the *J*–*V* curves and parameters of the corresponding PSCs were obtained, as presented in Fig. S6 and Table S1, respectively. The poor PCE value (2.5%) of those PSCs using a pure GO film can possibly be attributed to the scattered distribution of the GO pieces and the inferior conductivity of the GO due to the thinner GO sheet and high C–O content [28]. Those PSCs using the ethanol-treated PEDOT:PSS produced a relatively low PCE of 9.87%, which can be attributed to the inferior wettability of the PEDOT:PSS after ethanol treatment. Therefore, the scattered distribution of the pure GO hole extraction layer and ethanol-treated PEDOT:PSS is such that an efficient PSC cannot be attained. Figure 3b shows a cross-sectional image of PSCs with GO-modified PEDOT:PSS. The thicknesses of the CH_3_NH_3_PbI_3_ and PCBM layers are about 140 and 50 nm, respectively. Figure 3c compares the *J*–*V* curves of PSCs with and without GO-modified PEDOT:PSS, while Fig. 4 shows the averages and standard deviations for the characteristics of 30 devices. The optimized GO concentration in ethanol for the GO-modified PEDOT:PSS was 30 mg L^−1^. The best cells with and without the GO-modified PEDOT:PSS attained a PCE of 15.34% and 11.90%, respectively. The *J*_sc_, FF, and *V*_oc_ values of the PSCs with the GO modification are obviously higher than those of PSCs with only PEDOT:PSS, indicating that the use of the GO-modified PEDOT:PSS can significantly improve the performance of a PSC. The PCEs of cells with PEDOT:PSS modified with GO at a concentration of 10 and 50 mg L^−1^ are 13.44% and 14.82%, respectively, as presented in Fig. S7 and Table S2. No significant hysteresis phenomena were observed for cells fabricated with PEDOT:PSS, with and without the GO modification, as shown in Fig. 3c. The lower reverse saturation current and higher rectification ratio of those PSCs fabricated with the GO-modified PEDOT:PSS relative to PEDOT:PSS suggest that those PSCs using the GO-modified PEDOT:PSS can reduce the leakage current, inhibit the carrier recombination current, and improve the carrier transport and collection abilities [11, 14]. This can be partly attributed to the formation of a high-quality perovskite layer with better crystallinity and fewer pin holes due to the superior wettability of the GO-modified PEDOT:PSS. The partial removal of the low-conductivity PSS material at the PEDOT:PSS surface can improve contact properties between the perovskite and PEDOT:PSS layers, which helps to enhance the hole-collecting ability [13]. The hole-selective transport properties of the GO pieces can also improve the hole extraction and limit the recombination current from the electrons and holes [27, 30]. The UPS spectra of the PEDOT:PSS and GO-modified PEDOT:PSS layers are shown in Fig. 3d. The work function of the GO-modified PEDOT:PSS is 5.2 eV, which is higher than that of pristine PEDOT:PSS (4.9 eV). This enhanced work function improves the energy alignment between the ITO and the perovskite active layer, which reduces the energy loss of the hole transfer and increases the open-circuit voltage of those cells fabricated using the GO-modified PEDOT:PSS._ENREF_40

**Fig. 5:**
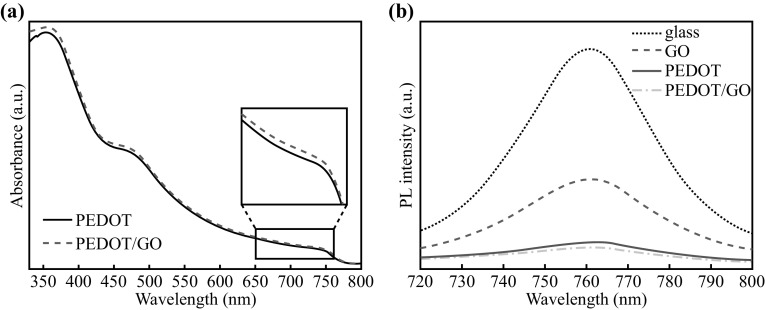
**a** UV–Vis absorption spectra of perovskite films fabricated on PEDOT:PSS layer modified with (*dash line*) and without (*solid line*) GO. **b** Photoluminescence of perovskite films on glass, GO/glass, PEDOT:PSS and GO-modified PEDOT:PSS (PEDOT/GO) substrates, respectively

The absorption spectra of the perovskite films formed on GO-modified and unmodified PEDOT:PSS are shown in Fig. 5a. The absorption spectra of the perovskite films exhibit an absorption onset at 790 nm and a shoulder band at 480 nm, which approximate to the results reported by Lee et al. [29]. The slightly enhanced absorption near the band gap edge of the perovskite film formed on the GO-modified PEDOT:PSS surface can usually be attributed to the superior crystallinity with fewer grain boundaries and surfaces, thus allowing more uniform crystallization [38]. Figure 5b shows the photoluminescence (PL) of perovskite films formed on different substrates. The PL intensities of the perovskite film deposited on PEDOT:PSS and GO-modified PEDOT:PSS layers are significantly quenched, indicating that the excitons of the perovskite films are efficiently separated and transferred into the hole transport layer. The lower relative PL intensity of the GO-modified perovskite film suggests that the GO modification can accelerate hole extraction from the perovskite layer to the hole transport layer, which supports the enhanced performance of those PSCs formed on the GO-modified PEDOT:PSS [28]. To further explain the mechanism of the enhanced performance of PSCs with the GO-modified PEDOT:PSS, the *J*–*V* characteristic curves were analyzed. For planar heterojunction solar cells, the *J*–*V* characteristic is usually expressed as [39–41]:

1 $$J = J_{{\rm L}} - J_{0} \left[{\exp \left({\frac{{e\left({V + JR_{{\rm S}}} \right)}}{{AK_{{\rm B}} T}}} \right) - 1} \right] - \frac{{V + JR_{{\rm S}}}}{{R_{{\rm sh}}}}$$

where *J*_L_ is the light-induced constant current density, *J* is the current density, *V* is the DC bias voltage, *J*_0_ is the dark saturated reverse current density of a PN heterojunction, *R*_s_ is the series resistance, *K*_B_ is the Boltzmann constant, *A* is the ideality factor, *e* is electron charge, *T* is the absolute temperature, and *R*_sh_ represents the shunt resistance. For a real device, Eq. 1 can be deduced as:

2 $$- \frac{{{\text{d}}V}}{{{\text{d}}J}} = \frac{{AK_{B} T}}{e}\left( {J_{\text{sc}} - J} \right)^{ - 1}\, +\, R_{\text{s}}$$

3 $$\ln \left( {J_{\text{sc}} - J} \right) = \frac{e}{{AK_{\text{B}} T}}\left( {V + JR_{\text{S}} } \right) + \ln J_{0}$$

**Fig. 6:**
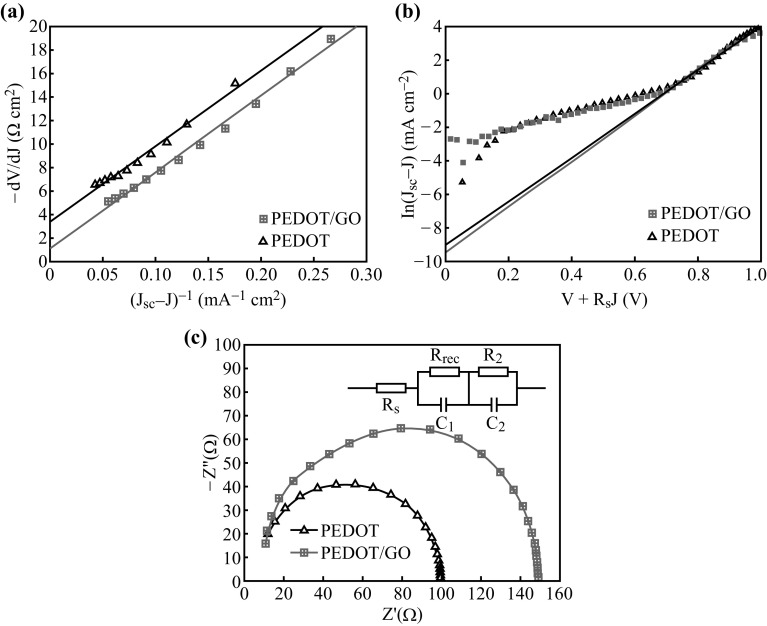
**a** Plots of −d*V/*d*J* versus *(J*_sc_ − *J*)^−1^ and **b** ln*(J*_sc_ − *J)* versus *(V* + *R*_s_ *J)* together with linear fitting of cells with and without GO modification under AM 1.5 illumination. **c** Nyquist plots of devices with and without GO modification with the forward bias voltage of 0.9 V in the dark

Figure 6a plots −d*V/*d*J* against *(J*_SC_ − *J*)^−1^ and the linear fitting curves obtained using Eq. 2. There is a good linear relationship between −d*V/*d*J* and *(J*_SC_ − *J*)^−1^, indicating that the fabricated cell is a well-behaved planar heterojunction solar cell. The intercepts of the fitting curves for the cells with and without the GO modification correspond to 1.08 and 3.37 Ω cm^−2^ of *R*_s_, respectively. The relatively low *R*_s_ contributes to the larger FF and *J*_sc_ of those cells with the GO-modified PEDOT:PSS [41]. Using Eq. 3, the *J*–*V* curves were rescaled and fitted, as shown in Fig. 6b. The value of *J*_0_ was determined to be 8.05 × 10^−5^ and 1.21 × 10^−4^ mA cm^−2^ for the devices with and without the GO modification.

Based on Eq. 1, the *V*_OC_ is obtained when *R*_sh_ is sufficiently large.

4 $$V_{\text{oc}} = \frac{{AK_{\text{B}} T}}{e}\ln \left( {\frac{{J_{\text{L}} }}{{J_{0} }} + 1} \right) \approx \frac{{AK_{\text{B}} T}}{e}\ln \left( {\frac{{J_{\text{sc}} }}{{J_{0} }}} \right)$$

Equation 4 indicates that a lower value of *J*_0_ corresponds to a higher value of *V*_OC_. Therefore, the cell with the GO modification exhibits a significantly higher value of *V*_OC_. As is well known, *J*_0_ represents the thermal emission rate of electrons from the valence band to the conduction band of the light absorber of the solar cells and is directly proportional to the recombination rate. Hence, the improved *V*_OC_ of the device with the GO modification can be attributed to the reduction in the recombination current from the holes and electrons [40]. The recombination resistances (*R*_rec_) can be obtained by fitting the Nyquist plots, which were measured from 1 Hz to 1 MHz at a forward bias voltage of 0.9 V in the dark with a model inserted in Fig. 6c. According to the equivalent circuit model, the series resistance is expressed as *R*_S_ and the recombination resistance and chemical capacitance are related to *R*_rec_ and *C*_2_, respectively. The *R*_1_ and *C*_1_ values are related to the selective contact or interface contact with the perovskite film layer_ENREF_45 The main low-frequency arc is caused by the recombination resistance of the interface. A larger arc indicates a larger recombination resistance and a decrease in the carrier recombination of those cells fabricated using the GO-modified PEDOT:PSS [30, 41, 42].

**Fig. 7:**
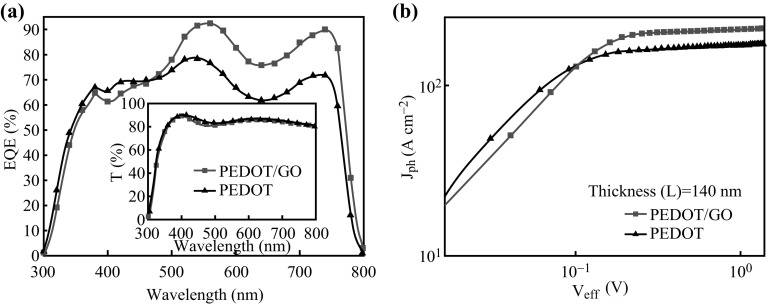
**a** IPCE spectra and **b** plots of *J*_ph_ versus *V*_eff_ for cells with and without GO modification. (*Inset*) Transmittance spectra of PEDOT:PSS and GO-modified PEDOT:PSS films

Figure 7a presents the incident photon to current conversion efficiency (IPCE) curves of PSCs. The slight decrease in the wavelength range from 400 to 500 nm can be attributed to the reduction in the light transmittance of the hole transport layer due to the existence of the GO. The clearly enhanced IPCE values in a wavelength range of 500–800 nm are usually attributed to the enhanced absorption and the stronger carrier transport and extraction abilities [11]. Figure 7b shows the effect of the GO on the photocurrent density (*J*_ph_) versus the effective voltage *(V*_eff_ *). J*_ph_ is determined as *J*_ph_ = *J*_light_ – *J*_dark_, where *J*_light_ and *J*_dark_ are the current density under illumination and in the dark, respectively. *V*_eff_ is determined as *V*_eff_ = *V*_0_ − *V*_a_, where *V*_0_ is the voltage at which *J*_ph_ = *0* and *V*_a_ is the applied bias voltage. *J*_ph_ increases linearly at a low *V*_eff_ range and saturates at a high value of *V*_eff_. In general, the saturated photocurrent (*J*_sat_) is correlated with the maximum exciton generation rate (*G*_max_), exciton dissociation probability, and carrier transporting and collecting probability when the value of *V*_eff_ is high [11, 43]. *G*_max_ could be obtained as *J*_ph_ = *qG*_max_ *L*, where *q* is the electronic charge and *L* is the thickness of the perovskite film (140 nm). The values of *G*_max_ for the devices with and without the GO modification are 9.58 × 10^27^ m^−3^ s^−1^ (*J*_sat_ = 214 A m^−2^) and 7.73 × 10^27^ m^−3^ s^−1^ (*J*_sat_ = 173 A m^−2^), respectively. The enhanced value of *G*_max_ reveals that those devices with the GO modification exhibit increased light absorption and decreased charge recombination at the grain boundaries and interface between the perovskite and PEDOT:PSS layers. The carrier transporting and collecting probability at any effective voltage can be obtained directly from the ratio of *J*_ph_/*J*_sat_. The carrier transporting and collecting probability of cells under the *J*_sc_ condition increased from 98.03% in the control device to 99.32% in a cell with the GO modification, indicating that the GO modification can improve the hole transportation and collection abilities of the cells.

**Fig. 8:**
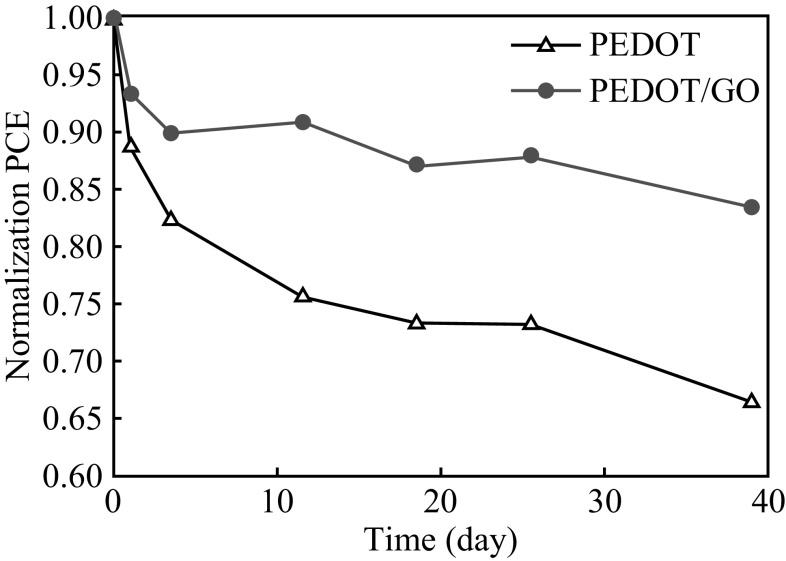
Normalized PCE decay averaged for 16 devices of each type, without encapsulation, after aging at RH 15% in air

The high-quality perovskite layer formed due to the superior wettability of the GO-modified PEDOT:PSS and the less hydrophilic PSS material at the interface between the perovskite and PEDOT:PSS both contribute to improving the moisture resistance of cells [2, 15]. Figure 8 shows the normalized PCE of the PSCs as a function of aging. The devices, without encapsulation, were kept in a drying cabinet with a relative humidity (RH) of 15% under the ambient atmosphere. The PCEs of PSCs fabricated with the GO-modified and unmodified PEDOT:PSS remained at 83.5 and 60% of the initial PCE values, respectively, after aging for 39 days, indicating that the GO modification improves the performance and stability of cells.

## Conclusions

In summary, the PCE of PSCs with the GO modification can be as high as 15.34%, which is obviously higher than that (11.90%) of PSCs fabricated with the unmodified PEDOT:PSS. The PCE of PSCs fabricated with the GO-modified PEDOT:PSS remains at 83.5% of the initial PCE value after aging for 39 days, indicating that the use of the GO-modified PEDOT:PSS instead of the unmodified PEDOT:PSS as a hole transport layer is a good strategy for efficiently improving the performance and stability of PSCs. The ethanol in the GO solution can partially remove the hydrophilic PSS material at the PEDOT:PSS surface during the spin-coating of the GO solution, which can improve the moisture resistance and decrease the contact barrier at the PEDOT:PSS surface. The scattered distribution of the GO pieces can further limit the hydrophobicity of the PEDOT:PSS surface due to the partial isolation of the GO pieces, which can also improve the moisture resistance. The superior wettability of the GO-modified PEDOT:PSS surface helps to form a high-quality perovskite layer with better crystallinity and fewer pin holes. Furthermore, the GO exhibits selectivity in the hole extraction, which can inhibit the recombination of holes and electrons at the interface between the perovskite and PEDOT:PSS. Therefore, the cooperative interactions of these factors can greatly improve the absorption of the perovskite layer, the carrier transport and collection abilities of PSCs, and especially the stability of the cells. Our results could form a basis for a strategy to overcome the weakness of PEDOT:PSS as a typical hole transport material and fully develop its advantages for application to perovskite solar cells.

## Electronic supplementary material

Below is the link to the electronic supplementary material.

Supplementary material 1 (PDF 1061 kb)

## References

[1] NREL Best Research-Cell Efficiencies (2016). http://www.nrel.gov/pv/assets/images/efficiency_chart.jpg. Accessed Sept 2016

[2] Salim T, Sun S, Abe Y, Krishna A, Grimsdale AC, Lam YM (2015). Perovskite-based solar cells: impact of morphology and device architecture on device performance. J. Mater. Chem. A.

[3] Zhang C, Luo Y, Chen X, Chen Y, Sun Z, Huang S (2016). Effective improvement of the photovoltaic performance of carbon- based perovskite solar cells by additional solvents. Nano-Micro Lett..

[4] Hou X, Xuan T, Sun H, Chen X, Li H, Pan L (2016). High-performance perovskite solar cells by incorporating a ZnGa_2_O_4_:Eu^3+^ nanophosphor in the mesoporous TiO_2_ layer. Sol. Energy Mater. Sol. Cells.

[5] You J, Hong Z, Yang Y, Chen Q, Cai M (2014). Low-temperature solution-processed perovskite solar cells with high efficiency and flexibility. ACS Nano.

[6] Todorov T, Gershon T, Gunawan O, Lee YS, Sturdevant C, Chang L-Y, Guha S (2015). Monolithic perovskite-CIGS tandem solar cells via in situ band gap engineering. Adv. Energy Mater..

[7] Luo P, Liu Z, Xia W, Yuan C, Cheng J, Lu Y (2015). Uniform, stable, and efficient planar-heterojunction perovskite solar cells by facile low-pressure chemical vapor deposition under fully open-air conditions. ACS Appl. Mater. Interfaces.

[8] Yang WS, Noh JH, Jeon NJ, Kim YC, Ryu S, Seo J, Seok SI (2015). High-performance photovoltaic perovskite layers fabricated through intramolecular exchange. Science.

[9] Liu X, Xia X, Cai Q, Cai F, Yang L, Yan Y, Wang T (2017). Efficient planar heterojunction perovskite solar cells with weak hysteresis fabricated via bar coating. Sol. Energy Mater. Sol. Cells.

[10] Huang L, Li C, Sun X, Xu R, Du Y (2017). Efficient and hysteresis-less pseudo-planar heterojunction perovskite solar cells fabricated by a facile and solution-saving one-step dip-coating method. Org. Electron..

[11] Jia X, Jiang Z, Chen X, Zhou J, Pan L, Zhu F, Sun Z, Huang S (2016). Highly efficient and air stable inverted polymer solar cells using LiF-modified ITO cathode and MoO_3_/AgAl alloy anode. ACS Appl. Mater. Interfaces.

[12] Jørgensen M, Norrman K, Krebs FC (2008). Stability/degradation of polymer solar cells. Sol. Energy Mater. Sol. Cells.

[13] Mengistie DA, Ibrahem MA, Wang P-C, Chu C-W (2014). Highly conductive PEDOT:PSS treated with formic acid for ITO-free polymer solar cells. ACS Appl. Mater. Interfaces.

[14] Jiang Z, Chen X, Lin X, Jia X, Wang J (2016). Amazing stable open-circuit voltage in perovskite solar cells using AgAl alloy electrode. Sol. Energy Mater. Sol. Cells.

[15] Adam G, Kaltenbrunner M, Głowacki ED, Apaydin DH, White MS (2016). Solution processed perovskite solar cells using highly conductive PEDOT:PSS interfacial layer. Sol. Energy Mater. Sol. Cells.

[16] Wang J, Xiang X, Yao X, Xiao W-J, Lin J, Li W-S (2016). Efficient perovskite solar cells using trichlorosilanes as perovskite/PCBM interface modifiers. Org. Electron..

[17] Ye S, Sun W, Li Y, Yan W, Peng H, Bian Z, Liu Z, Huang C (2015). CuSCN-based inverted planar perovskite solar cell with an average PCE of 15.6%. Nano Lett..

[18] You J, Meng L, Song T-B, Guo T-F, Yang Y (2016). Improved air stability of perovskite solar cells via solution-processed metal oxide transport layers. Nat. Nano.

[19] Christians JA, Fung RCM, Kamat PV (2014). An inorganic hole conductor for organo-lead halide perovskite solar cells. Improved hole conductivity with copper iodide. J. Am. Chem. Soc..

[20] Chen W, Wu Y, Yue Y, Liu J, Zhang W (2015). Efficient and stable large-area perovskite solar cells with inorganic charge extraction layers. Science.

[21] Wang Z-K, Li M, Yuan D-X, Shi X-B, Ma H, Liao L-S (2015). Improved hole interfacial layer for planar perovskite solar cells with efficiency exceeding 15%. ACS Appl. Mater. Interfaces.

[22] Park IJ, Park MA, Kim DH, Park GD, Kim BJ (2015). New hybrid hole extraction layer of perovskite solar cells with a planar p–i–n Geometry. J. Phys. Chem. C.

[23] Jiang Y, Li C, Liu H, Qin R, Ma H (2016). Poly(3,4-ethylenedioxythiophene):Poly(styrenesulfonate)(PEDOT:PSS)-molybdenum oxide composite films as hole conductor for efficient planar perovskite solar cells. J. Mater. Chem. A.

[24] Schniepp HC, Li J-L, McAllister MJ, Sai H, Herrera-Alonso M (2006). Functionalized single graphene sheets derived from splitting graphite oxide. J. Phys. Chem. B.

[25] Acik M, Darling SB (2016). Graphene in perovskite solar cells: device design, characterization and implementation. J. Mater. Chem. A.

[26] Palma AL, Cinà L, Pescetelli S, Agresti A, Raggio M, Paolesse R, Bonaccorso F, Di Carlo A (2016). Reduced graphene oxide as efficient and stable hole transporting material in mesoscopic perovskite solar cells. Nano Energy.

[27] Li S-S, Tu K-H, Lin C-C, Chen C-W, Chhowalla M (2010). Solution-processable graphene oxide as an efficient hole transport layer in polymer solar cells. ACS Nano.

[28] Wu Z, Bai S, Xiang J, Yuan Z, Yang Y (2014). Efficient planar heterojunction perovskite solar cells employing graphene oxide as hole conductor. Nanoscale.

[29] Lee D-Y, Na S-I, Kim S-S (2016). Graphene oxide/PEDOT:PSS composite hole transport layer for efficient and stable planar heterojunction perovskite solar cells. Nanoscale.

[30] Li W, Dong H, Guo X, Li N, Li J, Niu G, Wang L (2014). Graphene oxide as dual functional interface modifier for improving wettability and retarding recombination in hybrid perovskite solar cells. J. Mater. Chem. A.

[31] Cote LJ, Kim F, Huang J (2009). Langmuir–blodgett assembly of graphite oxide single layers. J. Am. Chem. Soc..

[32] Manga KK, Zhou Y, Yan Y, Loh KP (2009). Multilayer hybrid films consisting of alternating graphene and titania nanosheets with ultrafast electron transfer and photoconversion properties. Adv. Funct. Mater..

[33] de Jong MP, van Ijzendoorn LJ, de Voigt MJA (2000). Stability of the interface between indium-tin-oxide and poly(3,4-ethylenedioxythiophene)/poly(styrenesulfonate) in polymer light-emitting diodes. Appl. Phys. Lett..

[34] Alemu D, Wei H-Y, Ho K-C, Chu C-W (2012). Highly conductive PEDOT:PSS electrode by simple film treatment with methanol for ITO-free polymer solar cells. Energy Environ. Sci..

[35] Zhang S, Yu Z, Li P, Li B, Isikgor FH (2016). Poly(3,4-ethylenedioxythiophene):polystyrene sulfonate films with low conductivity and low acidity through a treatment of their solutions with probe ultrasonication and their application as hole transport layer in polymer solar cells and perovskite solar cells. Org. Electron..

[36] Wu Y, Islam A, Yang X, Qin C, Liu J, Zhang K, Peng W, Han L (2014). Retarding the crystallization of PbI2 for highly reproducible planar-structured perovskite solar cells via sequential deposition. Energy Environ. Sci..

[37] Wang L, McCleese C, Kovalsky A, Zhao Y, Burda C (2014). Femtosecond time-resolved transient absorption spectroscopy of CH_3_NH_3_PbI_3_ perovskite films: evidence for passivation effect of PbI_2_. J. Am. Chem. Soc..

[38] D’Innocenzo V, Srimath Kandada AR, De Bastiani M, Gandini M, Petrozza A (2014). Tuning the light emission properties by band gap engineering in hybrid lead halide perovskite. J. Am. Chem. Soc..

[39] Shi J, Dong J, Lv S, Xu Y, Zhu L (2014). Hole-conductor-free perovskite organic lead iodide heterojunction thin-film solar cells: high efficiency and junction property. Appl. Phys. Lett..

[40] You J, Yang Y, Hong Z, Song T-B, Meng L (2014). Moisture assisted perovskite film growth for high performance solar cells. Appl. Phys. Lett..

[41] Zhu W, Bao C, Wang Y, Li F, Zhou X (2016). Coarsening of one-step deposited organolead triiodide perovskite films via Ostwald ripening for high efficiency planar-heterojunction solar cells. Dalton Trans..

[42] Yu H, Roh J, Yun J, Jang J (2016). Synergistic effects of three-dimensional orchid-like TiO_2_ nanowire networks and plasmonic nanoparticles for highly efficient mesoscopic perovskite solar cells. J. Mater. Chem. A.

[43] Xu M-F, Zhu X-Z, Shi X-B, Liang J, Jin Y, Wang Z-K, Liao L-S (2013). Plasmon resonance enhanced optical absorption in inverted polymer/fullerene solar cells with metal nanoparticle-doped solution-processable TiO_2_ layer. ACS Appl. Mater. Interfaces.

