# Supplementary material for: Efficient and Air-Stable Planar Perovskite Solar Cells Formed on Graphene-Oxide-Modified PEDOT:PSS Hole Transport Layer
Source: Nanomicro Lett. 2017 Mar 17;9(4):39. doi: 10.1007/s40820-017-0140-x (PMC6199037; doi:10.1007/s40820-017-0140-x)
Supplement: Supplementary file 1 — Supplementary material 1 (PDF 1061 kb) [file 40820_2017_140_MOESM1_ESM.pdf]

Supporting Information for

Efficient and Air Stable Planar Perovskite Solar Cells via Graphene Oxide Modified PEDOT:PSS Hole Transport Layer

Hui Luo[1], Xuanhuai Lin[1], Xian Hou[1], Likun Pan[1], Sumei Huang[1], Xiaohong Chen[1,*]

[1]Engineering Research Center for Nanophotonics & Advanced Instrument, Ministry of Education, and School of Physics and Materials Science, East China Normal University, No. 3663 North Zhongshan Rd., Shanghai 200062, People's Republic of China

* Corresponding author. E-mail: xhchen@phy.ecnu.edu.cn

Tel.:+86 21 62233676, Fax: +86 21 62234321

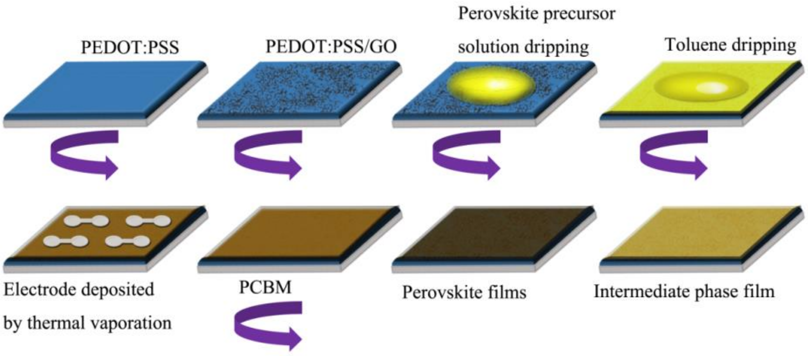

Fig. S1 Schematic illustration of engineering process of cells with GO modified PEDOT:PSS layer

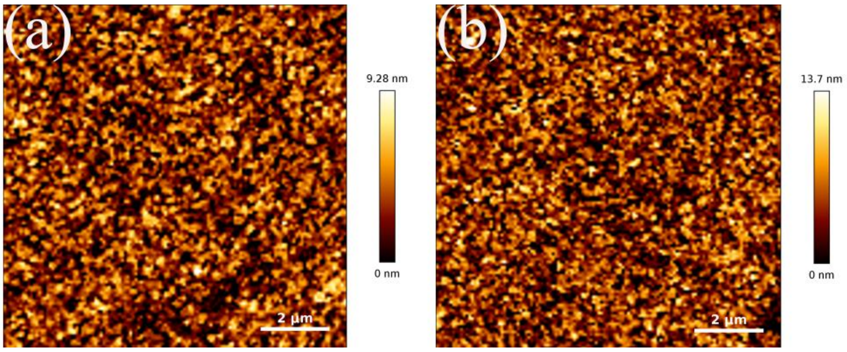

Fig. S2 AFM images of PEDOT:PSS modified without **a** and with **b** GO pieces

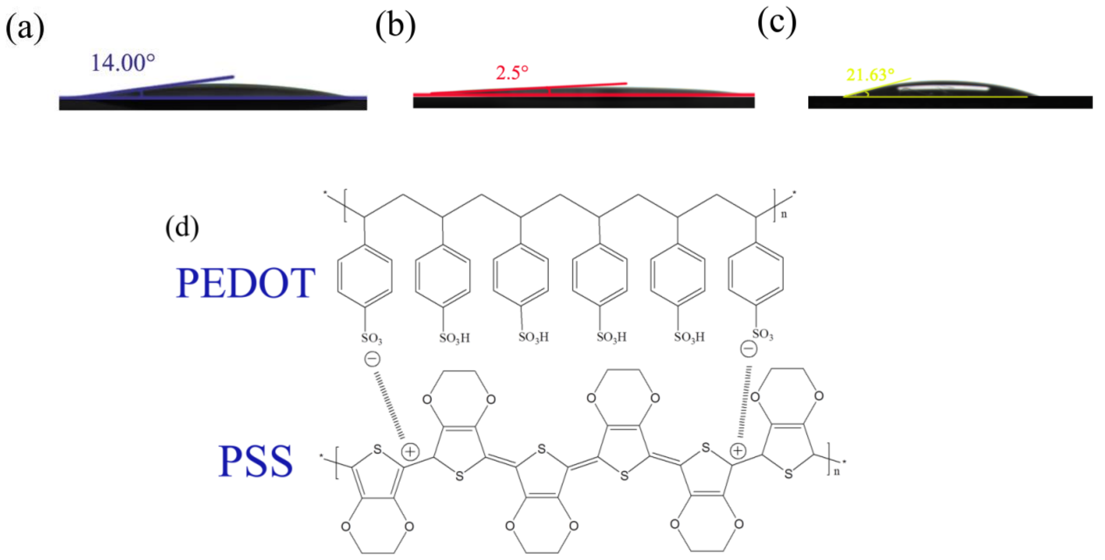

Fig. S3 Contact angles of PEDOT:PSS surface modified with different condition. **a** PEDOT:PSS surface; **b** GO modified PEDOT:PSS surface; **c** PEDOT:PSS with ethanol treatment; **d** Chemical structure of PEDOT:PSS

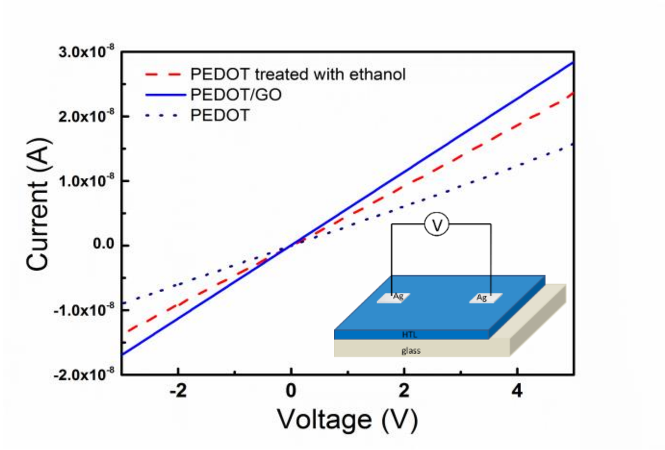

Fig. S4 *I–V* curves of PEDOT:PSS, PEDOT:PSS treated with ethanol and PEDOT:PSS/GO films and the inset is the schematic diagram of testing structure

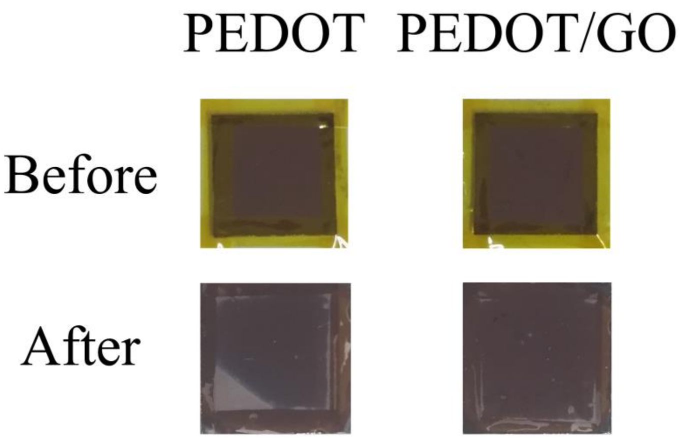

Fig. S5 Images of perovskite films growth on PEDOT:PSS and GO modified PEDOT:PSS before and after exposed to the saturated water vapor pressure at room temperature for 1.5 h

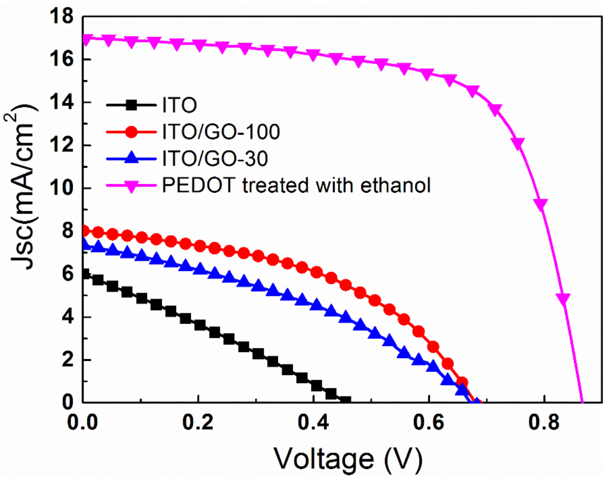

Fig. S6 *J–V* curves of PSCs with different hole transport layer. Perovskite layer was directly spin coated onto ITO, ITO/GO-30, ITO/GO-100 and PEDOT:PSS treated with ethanol solvent, respectively. GO concentrations spin-coated onto ITO electrode are 30 mg L^{-1} (GO-30) and 100 mg L^{-1} (GO-100), respectively. PEDOT:PSS surface after annealing at 140 °C was treated with ethanol at 4000 rpm for 1 min

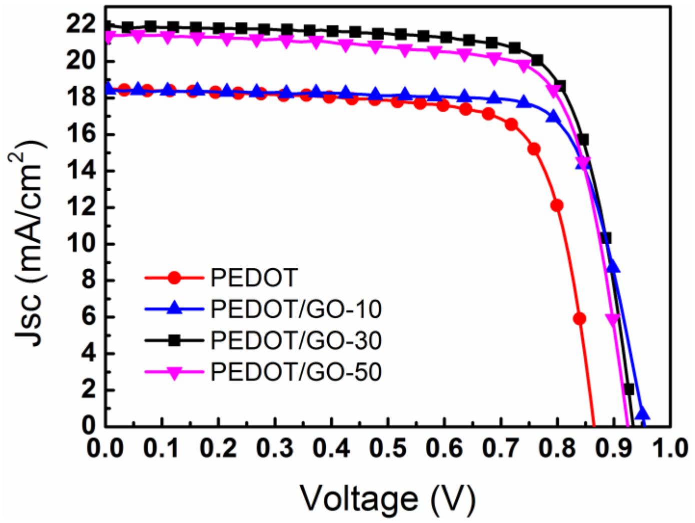

Fig. S7 *J–V* curves of PSCs with different hole transport layer: PEDOT:PSS and PEDOT:PSS modified with GO of 10 mg L^{-1} (GO-10), 30 mg L^{-1} (GO-30), 50 mg L^{-1} (GO-50) concentration

Table S1 Parameters of corresponding PSCs from Fig. S3

**Hole transport layer**	**J_{sc} (mA cm^{-2})**	**V_{oc} (V)**	***FF* (%)**	***PCE* (%)**
ITO/GO-30	7.34	0.68	36.70	1.83
ITO/GO-100	8.02	0.68	45.92	2.51
PEDOT:PSS with ethanol treatment	17.00	0.87	67.09	9.87
ITO	6.01	0.46	27.41	0.76

Table S2 Parameters of corresponding PSCs from Fig. S4

Hole transport layer	V_{oc} (V)	J_{sc} (mA cm^{-2})	*FF* (%)	*PCE* (%)
PEDOT	0.87	18.47	74.12	11.90
PEDOT/GO-10	0.95	18.43	76.40	13.44
PEDOT/GO-30	0.94	21.92	74.78	15.34
PEDOT/GO-50	0.92	21.47	74.67	14.82
